# “Doing Things Together Is What It’s About”: An Interpretative Phenomenological Analysis of the Experience of Group Therapeutic Songwriting From the Perspectives of People With Dementia and Their Family Caregivers

**DOI:** 10.3389/fpsyg.2021.598979

**Published:** 2021-03-31

**Authors:** Imogen N. Clark, Felicity A. Baker, Jeanette Tamplin, Young-Eun C. Lee, Alice Cotton, Phoebe A. Stretton-Smith

**Affiliations:** ^1^Melbourne Conservatorium of Music, Faculty of Fine Arts and Music, The University of Melbourne, Melbourne, VIC, Australia; ^2^Centre for Research in Music and Health, Norwegian Academy of Music, Oslo, Norway; ^3^Austin Health, The University of Melbourne, Melbourne, VIC, Australia

**Keywords:** group therapeutic songwriting, music therapy, people with dementia/family caregiver dyads, personhood, couplehood, family centeredness, interpretative phenomenological analysis

## Abstract

**Background:**

The wellbeing of people living with dementia and their family caregivers may be impacted by stigma, changing roles, and limited access to meaningful opportunities as a dyad. Group therapeutic songwriting (TSW) and qualitative interviews have been utilized in music therapy research to promote the voices of people with dementia and family caregivers participating in separate songwriting groups but not together as dyads.

**Procedures:**

This study aimed to explore how ten people with dementia/family caregiver dyads experienced a 6-week group TSW program. Dyads participated in homogenous TSW groups involving 2–4 dyads who were either living together in the community (2 spousal groups) or living separately because the person with dementia resided in a care home (1 family group, 1 spousal group). The TSW program, informed by personhood, couplehood, family centered and group process frameworks, involved creating original lyrics through song parody and song collage. Qualified Music Therapists facilitated sessions and interviewed each dyad separately. Interviews were analyzed using interpretative phenomenological analysis.

**Findings:**

Five recurrent group themes were developed, indicating group TSW: (1) was a positive shared experience, benefiting both members of the dyad and motivating further engagement with music; (2) stimulated mental processes and reignited participants’ interests and skills; (3) provided meaningful opportunities for reflection and connection with memories and life experiences; and (4) prompted interaction and collaboration, leading to social connections, empathic relationships and experiences of inclusion. Participants also highlighted how: (5) the facilitated process supported engagement, highlighting abilities and challenging doubts.

**Conclusion:**

Dyads identified group TSW as an opportunity to recognize strengths, voice ideas and opinions, share meaningful experiences, and do “more with music.” Participants valued TSW as a new, creative and stimulating experience that enabled connection with self and others and led to feelings of pride and achievement. Our findings further recognize how therapeutic intention and approach were reflected in participants’ engagement and responses regardless of dementia stage and type, dyad relationship, or musical background. This research may broaden perspectives and expand understanding about how people with dementia and their family caregivers access and engage in music therapy.

## Introduction

The health and wellbeing of people with dementia and their family caregivers is highly interconnected, requiring acknowledgment of unique individual and shared relational lived experiences (McGillick and Murphy-White, 2016). Research has attempted to elucidate the lived experiences of people with dementia (Wolverson et al., 2016; Low et al., 2017) and family caregivers (McCabe et al., 2016; Egilstrod et al., 2019; Hennings and Froggatt, 2019), although the perspectives of people with dementia have received less research attention (Thompson et al., 2020). Findings suggest that people with dementia seek to maintain or adjust self-identity, engage in meaningful activities, and continue personal growth, while contending with experiences of public- and self-stigma and concerns about lack of control in the present and future (Milne, 2011; Wolverson et al., 2016; Low et al., 2017; Nguyen and Li, 2020). Family caregivers, whether cohabitating or living separately because their family member with dementia resides in a care home, also recognize concerns about the future, managing role and relationship changes, and meeting their own personal, physical and psychological health and wellbeing needs (McCabe et al., 2016; Egilstrod et al., 2019; Hennings and Froggatt, 2019). Importantly, both people with dementia and family caregivers recognize the impact of social interaction, partnerships and connection on sense of self and wellbeing, highlighting the need for social support, meaningful relationships, and empathic group experiences (Milne, 2011; Hennings and Froggatt, 2019).

Recently, there has been an increasing awareness of people with dementia and family caregiver dyads’ shared and dynamic relational experiences (Ablitt et al., 2009; Wadham et al., 2016; Conway et al., 2018). The concept of “couplehood” (Hellström et al., 2007) builds on the seminal work of Kitwood (1997) and Keady (1999) and acknowledges how couples work together in interdependent, reciprocal and complementary ways to sustain their relationship and each other’s active engagement in meaningful life experiences (Hellström et al., 2007). This focus has extended to “family centeredness” in dementia care, a concept that also has roots in person-centered care (Hao and Ruggiano, 2020). The family centered care model encourages mutual support among families, considers quality of life of the family unit, and recognizes the valuable contributions of both the person receiving care and families providing care (Hao and Ruggiano, 2020). These models acknowledge both individual and centralized voices of people with dementia and their family caregivers as experts in their personal and shared experiences. Qualitative research reviews exploring shared experiences of dyads further recognize the need for dyad-based psychosocial interventions that provide opportunities for teamwork and meaningful joint experiences (Ablitt et al., 2009; Wadham et al., 2016; Conway et al., 2018; Hao and Ruggiano, 2020).

Music-based psychosocial interventions with people with dementia and their family caregivers offer accessible opportunities for active participation and enjoyment, experiences of mutuality and togetherness, and social connection with others in a similar situation (McDermott et al., 2013; Unadkat et al., 2017; Clark et al., 2018; Tamplin and Clark, 2019). Music is a powerful stimulus of autobiographical memories for people with dementia that is linked closely with self-identity and life history, and supports emotional expression and relationship-building (McDermott et al., 2014; Cuddy et al., 2015). As people with dementia are often able to access musical knowledge and skills using intact implicit or procedural musical memory, music-based interventions can also offer opportunities for experiences of leadership, achievement, and success (Spiro, 2010; Jacobsen et al., 2015), as well as alternative forms of self-expression and connection when verbal communication is difficult or confronting (McDermott et al., 2013). The psychosocial model of music in dementia, developed through thematic analysis of interviews with people with dementia, their family members, care home staff, and music therapists, further explains how music supports the personal psychology of people with dementia and social psychology of their care environment, leading to positive ripple effects among family and formal caregivers (McDermott et al., 2014). This psychosocial potential of music is enhanced in music therapy contexts by the client-therapist relationship and ability of trained music therapists to meet social, emotional, cultural, and personal needs of individuals with dementia (McDermott et al., 2013).

Research on dyad-based music therapy interventions with people with dementia and their family caregivers has been an evolving process. Early research examining the effects of active music therapy interventions suggested symptoms of dementia were reduced and family caregiver satisfaction was improved in dyads living together (Brotons and Marti, 2003) and separately when the person with dementia resided in a care home (Clair and Ebberts, 1997). Other dyad-based research exploring family caregiver-delivered music interventions guided by music therapists found that dyads who regularly shared music together experienced enhanced mutual engagement, relationship quality, and emotional wellbeing, as described by family caregivers of people with dementia in both community (Hanser et al., 2011; Baker et al., 2012) and residential care contexts (Clair, 2002). More recent research has captured the perspectives of both participants with dementia and their family caregivers who attended community-based therapeutic singing groups together (Clark et al., 2018; Tamplin et al., 2018). Findings recognized the complex interactions and affinity within and between dyads in the group, encompassing enjoyment of therapeutic group singing as a mutually meaningful experience, leading to development of empathic friendships, and individual experiences of happiness and pride. Importantly, dyads in this study highlighted feelings of togetherness and mutual benefits from being able to attend together. In the evolution of dyad-based music therapy research, we can see development from a medical focus on deficit and burden (Clair and Ebberts, 1997; Brotons and Marti, 2003), through to a focus on relational needs from the perspectives of caregivers (Clair, 2002; Hanser et al., 2011; Baker et al., 2012), and more recently a recognition of dyad mutuality and shared experiences among people with dementia and their family caregivers (Clark et al., 2018; Tamplin et al., 2018).

Therapeutic songwriting (TSW) is a music therapy intervention that has been used in research with people with dementia (Silber and Hes, 1995; Hong and Choi, 2011; Baker and Stretton-Smith, 2017) and family caregivers of people with dementia (Baker and Yeates, 2018; Baker et al., 2018; García-Valverde et al., 2019). Group TSW provides a forum to express and be heard with others who have similar life experiences and understandings (Baker, 2015). As a task-orientated process that often occurs over several sessions, opportunities for teamwork, social connection, and personal exploration may be intensified in songwriting groups (Baker, 2015). Baker (2015) recognized three group TSW approaches: (1) insight-orientated involving the exploration of feelings to develop insight and reconcile conflicting internal emotions; (2) narrative or storytelling as an opportunity to process feelings about the past, present, and future; and (3) psycho-educational songwriting as a way to shift thinking and identify strategies to deal with stressors. To date, research has explored TSW with separate groups attended by either people with dementia or family caregivers, but there does not appear to be any studies where people with dementia and their family caregivers participated in TSW groups together as dyads.

Based on findings from systematic reviews, TSW has received less attention than other music therapy methods with people with dementia (McDermott et al., 2013; Fusar-Poli et al., 2018; van der Steen et al., 2018; Li et al., 2019), possibly due to an assumption that songwriting may be too cognitively demanding. However, existing studies have challenged these assumptions. Early research by Silber and Hes (1995) involved community-dwelling people with dementia who attended weekly group sessions creating new lyrics to existing and unknown melodies. Findings based on researcher observations, suggested that group TSW stimulated preserved memory, creative processes, robust social discussion and expression of previously repressed emotions (Silber and Hes, 1995). A randomized controlled trial by Hong and Choi (2011) also found that people with dementia living in residential care who participated in a 16-week TSW program had improved pre to post overall cognition, language, orientation, and memory when compared to usual care. Finally, Baker and Stretton-Smith (2017) used interpretative phenomenological analysis of interviews to explore the experiences of four people with dementia and support staff who attended a 10-week group TSW program at a dementia care day center. Both participants with dementia and staff described TSW as a creative, collaborative and motivating experience that highlighted ability and led to enhanced feelings of social connection, belonging, confidence, and achievement. Further, while participants recognized TSW as a new and challenging process, they welcomed opportunities for mental stimulation and learning. This preliminary research suggests that group TSW may offer many social, emotional, cognitive, and psychological benefits for people with dementia living at home and in residential aged care.

Group TSW with family caregivers of people with dementia offers an alternative mode to traditional counseling where participants work together to explore personal identity, express emotions and feelings, and identify supportive strategies (Baker, 2017). Klein and Silverman (2012) used a psycho-educational approach to compare TSW and discussion groups with family caregivers of people with dementia and found that, while both interventions led to an improved understanding about self-care, TSW was more “fun” and enjoyable. Recent quantitative research found that 6 and 12 week group TSW programs resulted in moderate to large effects, suggesting that depression (Baker et al., 2018; García-Valverde et al., 2019) and anxiety (García-Valverde et al., 2019) might be reduced with fully powered samples. Further, analysis of qualitative interview data recognized how group TSW was different to traditional carer support groups as it provided valuable opportunities to explore and voice positive feelings, as well as challenges, around the “whole carer journey,” leading to the acknowledgment of personal strengths and growth (Baker et al., 2018).

While it is clear that group TSW offers benefits for people with dementia and their family caregivers attending separate groups, to our knowledge, there is no research examining group TSW with people with dementia and their family caregivers participating together as dyads. The current study aimed to address this gap, while building on existing dyad-based music therapy research. We also aimed to promote the voices of research participants by answering the question: how do people with dementia and their family caregivers describe their experiences of participating in a group TSW program? Focus was given to dyads’ experiences of participating together and as individuals within a group context. This qualitative enquiry took place alongside a feasibility study testing the acceptability of the intervention and sensitivity and appropriateness of outcome measures (Clark et al., 2020).

## Procedures

This paper reports interpretative phenomenological analysis of interviews conducted with dyads who completed a quasi-experimental pre–post feasibility trial examining a 6-week group TSW program (Clark et al., 2020). The project was funded by the Dementia Australia Research Foundation (Hazel Hawke Research and Dementia Care Grant) with approval from the University of Melbourne Human Ethics Committee (Ethics I.D. 1851252.1). All participants either signed informed consent or, if participants were unable to provide written consent because of advanced dementia, verbal assent was sought, and signed consent was provided by a family member with power of attorney who was not participating in the study.

### Epoché

An epoché was developed prior to the analysis of interviews by authors 1 and 6 to depict the researchers’ conscious identification and bracketing off of predetermined thoughts and ideas related to group TSW with people with dementia and their family caregivers (Hiller, 2016). Author 1 has practiced music therapy for 13 years and nursing for 20 years. Her clinical work and research have broadly focused on various music therapy methods with people with dementia and their family caregivers in community and healthcare contexts. Through this work, she has developed the preconceptions that people with dementia and their family caregivers may: (1) benefit both as individuals and dyads from participation in group TSW together; (2) value the socially and mentally stimulating aspects of group TSW; (3) experience mutuality following participation in group TSW. Author 6 has practiced as a music therapist for 3 years, including with people with dementia in community, aged care and inpatient contexts. She has also been involved in research with people with dementia and family caregivers participating in separate TSW groups. Through this clinical and research experience, the following preconceptions were developed: (1) people with dementia may find TSW challenging, novel and/or rewarding; (2) family caregivers may value TSW as an opportunity for self-expression; (3) both people with dementia and family caregivers may describe group TSW as a positive opportunity for socialization and connection.

### Participants

Dyads were recruited for the primary feasibility study through partnering not-for-profit organizations providing community support and residential care for older adults in Melbourne, Australia. Participants were eligible if they had functional hearing (with or without aids) and were aged 18 years or older. Participation was not restricted by dementia type, dyad relationship or living situation (Clark et al., 2020). Dyads who completed the TSW intervention were invited to participate in interviews.

**FIGURE 1:**
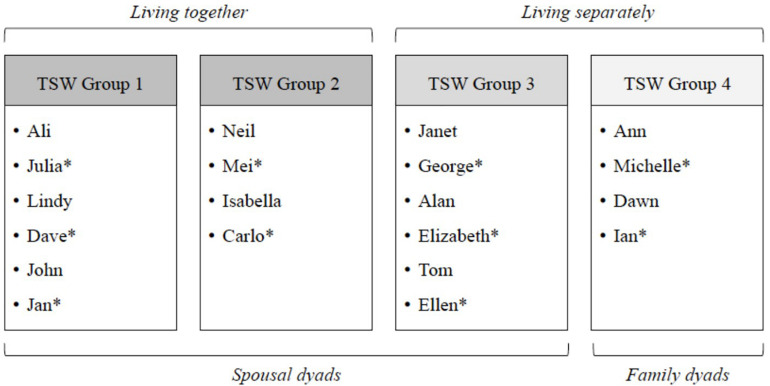
Songwriting groups.

The primary study included four homogenous TSW groups, which were formed based on dyads’ location, relationship, and living arrangement, including spousal dyads living together in their own homes (groups 1 and 2), and spousal (group 3) and family (group 4) dyads living separately as the person with dementia resided in a care home (see Figure 1, Songwriting groups). From the ten dyads (20 participants) who completed the TSW intervention, nine people with dementia and all family caregivers agreed to participate in interviews. One family caregiver was interviewed alone as their spouse was unable to participate due to advanced dementia. One participant with dementia who was interviewed used a supportive communication device. For participants with dementia living in residential care facilities, time since moving into care ranged from 6 to 24 months (*M* = 14 months) (see Table 1, Participant characteristics).

**TABLE 1:** Participant characteristics.

	Participants with dementia	Family caregivers
Female (male), *n*	4 (5)	6 (4)
Age in years*	77 (10.9), 62–92	70 (9.8), 59–92
**Country of birth, *n***		
Australia	8	7
Italy	–	1
Lebanon	1	1
Malaysia	–	1
MMSE/30*	14.1 (8.8), 0–29	–
Years since diagnosis*	3.6 (2.2), 1–9	–
**Diagnosis, *n***		
FTD	2	–
AD	2	–
AD/Vasc	3	–
STML	2	–

* Mean (SD), Range. MMSE, Mini Mental State Exam; FTD, Frontal Temporal Dementia; AD, Alzheimer’s Dementia; Vasc, Vascular Dementia; STML, Short Term Memory Loss.

Before beginning the program, participants were asked to describe their musical backgrounds during a music therapy assessment. Musical backgrounds were diverse, ranging from enjoyment of music listening, concerts and musicals, to histories of formal music tuition, choir singing, piano playing and ballroom dancing. Most participants had music participation experience from childhood and adolescence, although one participant with dementia had taken piano lessons as a memory strategy following diagnosis, and three participants were actively involved in music at the time of the study through singing in a therapeutic choir for people with dementia and their family members and playing piano at church.

### TSW Theoretical Approach and Intervention

**FIGURE 2:**
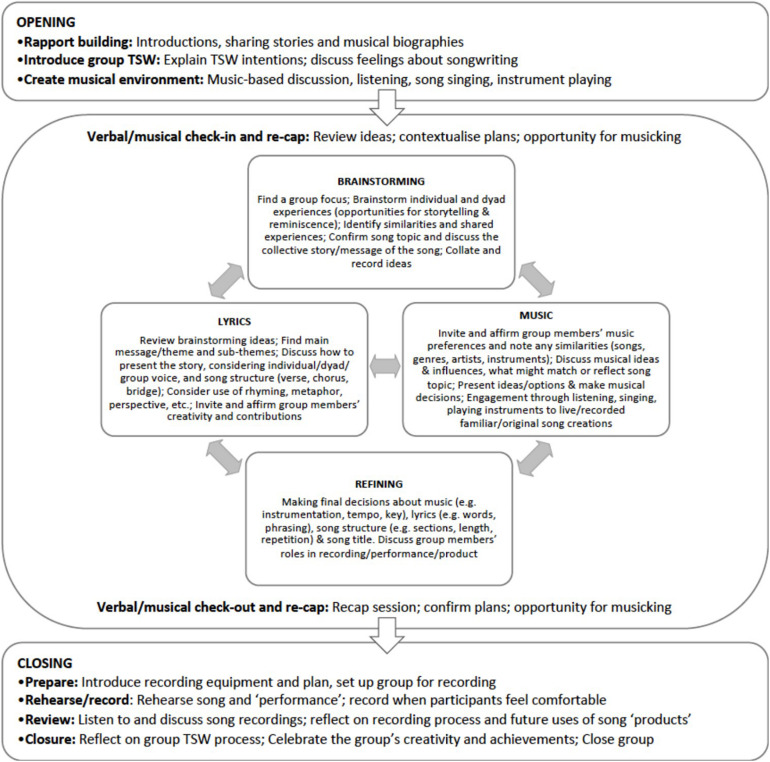
Session design.

As there is no existing research on group TSW with people with dementia and their family caregivers participating as dyads, and the inclusion criteria for this study was broad, a theoretical approach to TSW was developed to meet the unique needs and promote the diverse strengths and contributions of participants. This approach aimed to recognize individuals while also supporting partnerships at multiple levels, from within the dyad to those developed within and outside the songwriting group. To do this, we drew on concepts of personhood (Kitwood, 1997), couplehood (Hellström et al., 2007), family centeredness (Hao and Ruggiano, 2020), and group process (Yalom and Leszcz, 2005), as well as notions from community music therapy (Stige and Aarø, 2012). Facilitation of TSW groups was driven by participants and adapted to flexibly meet the strengths and resources of individuals, dyads and the group (see Figure 2, Session design).

The TSW program involved six 1-h sessions facilitated by a qualified music therapist (Author 5 or 6). Sessions were weekly, aside from a 2-week break over the end of year period, and attendance was high (*M* = 5.3 of 6 sessions, 88%). Sessions took place in private rooms at community spaces accessed through the partnering organization or the residential aged care facility where participants with dementia were living. Equipment included a whiteboard and markers, song books and lyric sheets, guitar, percussion instruments, Bluetooth speaker, and a laptop, microphones, headphones, and software for the recording session. Author 1 (an experienced music therapist who conceptualized the study) provided weekly supervision to both music therapists who facilitated the TSW groups. Supervision involved watching video recordings of sessions, discussing group responses and any challenges related to therapeutic facilitation and the songwriting process. Each group created and recorded two to three songs over the 6-week period, choosing to engage predominantly through the method of song parody by rewriting lyrics to familiar pre-composed songs (Baker, 2015) (see Table 2, description of group song creations).

**TABLE 2:** Description of group song creations.

Grp	Song title	Song topic	TSW method	Time	Music (duration mins)
1	Beauty of Nature	Being in nature – dyad stories, group experience	Song parody	4 sessions	Yellow Submarine (3:01)
	We Wish You a Merry Christmas	Reflections on past year and meaning of Christmas time	Song parody	1 session	We Wish You a Merry Christmas (2:26)
2	Dance Around the House Tonight	Social gatherings, group members’ personalities	Song parody	2 sessions	Rock Around the Clock (1:32)
	Star of the South	Memories of growing up, living in Melbourne	Song collage/original lyrics	3 sessions	Goodbye Yellow Brick Road/Love Me Tender (2:11)
3	Happy Days	Dyads’ shared pastimes, experience of reminiscence	Song parody	2 sessions	Waltzing Matilda (2:48)
	Memories of Home	“Home,” memories from childhood and adolescence	Song parody	3 sessions	Country Roads (3:07)
4	It’s Not Unusual	Memories of going out to social dances	Song parody	2.5 sessions	It’s Not Unusual (2:30)
	Picnic Van (Singing Along the Way)	Memories of picnics with family and friends	Song parody	1.5 sessions	Morningtown Ride (3:14)
	Memories	Reflection on TSW, music and memory	Song parody	1 session	Edelweiss (1:48)

### Data Collection

Data for this study were collected through semi-structured dyadic interviews. Interviews took place at a convenient time 1–2 weeks following the final TSW session at either the dyad’s home, the residential care facility where participants with dementia lived, or the same location where sessions were held. Interviews were conducted by the same music therapist who facilitated TSW sessions with those dyads. These decisions were made with consideration for both participants and the research process, and were informed by previous qualitative music therapy research exploring the experiences of people with dementia and dyads (McDermott et al., 2014; Baker and Stretton-Smith, 2017; Clark et al., 2018), as well as emerging research on inclusive qualitative data collection practices to support participants with dementia (Thompson et al., 2020). Familiarity with the space and interviewer was utilized to create a positive environment where participants felt comfortable and able to tell their stories. Dyadic rather than one-on-one interviews meant family caregivers were able to support their family member to expand and clarify or develop ideas in addition to contributing their own thoughts, feelings and perspectives. The dual role of music therapist/interviewer ensured existing rapport with the dyad and knowledge of how the person with dementia preferred to interact and communicate. The interviewer was also able to draw on therapeutic and musical skills and knowledge of the song creations to support and prompt participants.

Interview questions, formulated to garner perspectives on how dyads experienced the TSW group, asked: What was it like to work with others to write a song? What was it like to attend together as a dyad? Were the songwriting sessions as anticipated? What (if anything) stood out or was meaningful? How (if at all) was this group similar or different to other group experiences attended together? And, how (if at all) the songwriting sessions influenced the dyad’s interactions or relationship with each other? For the purpose of the associated feasibility study, participants also commented on whether they found the group TSW process worthwhile and if there were any changes they would recommend (Clark et al., 2020). Audio recorded interviews were 12–32 min in length (*M* = 22 min) for dyadic interviews, and 21 min in length for the interview that took place with the family caregiver only.

### Data Analysis

Authors 1 and 6 transcribed and then analyzed interviews using interpretative phenomenological analysis, drawing on frameworks, strategies and processes described by Smith et al. (2009) to explore, understand and communicate the experiences and perspectives of participants. Collaboration and dialog between authors at multiple points in the analysis process helped to “test and develop the coherence and plausibility of the interpretation” (Smith et al., 2009, 80) both at the single interview level (developing superordinate themes for each dyad) and group level (developing recurrent themes and sub-themes across cases), as well as during the write-up stage.

Analysis began with both authors listening to audio recordings, and reading and rereading interview transcripts to familiarize themselves with the data and establish participants as the focus of analysis. For the next step of initial noting and exploratory comments, both authors separately identified and examined participants’ descriptions, use of language, and any similarities, differences, repetitions, amplifications or ambiguities within their account. This step also included conceptual annotations, working to move away from the descriptive to the interpretative and open up “a range of provisional meanings” (Smith et al., 2009, 89). In developing emergent themes, each author narrowed their focus, identifying what was important and attempting to “capture and reflect an understanding” (p. 92) through themes that encompassed both participants’ own words and the authors’ interpretation. Authors 1 and 6 then discussed the interpretation of dyads’ described experiences and developed super-ordinate themes, which were formed using various strategies, including abstraction (identifying patterns and grouping like themes), subsumption (using an emergent theme as a superordinate theme to bring together related ideas), and polarization (identifying oppositional relationships between emergent themes). This whole process was repeated for each interview before looking for patterns across cases.

Cross-case analysis conducted by Authors 1 and 6 began using hard copy material, moving and arranging themes on a large piece of paper to explore “spatial representations” (Smith et al., 2009, 96) of how themes related to each other. Groups of related themes were then arranged in a word document and developed into recurrent group themes and sub-themes through abstraction. The arrangement of themes was extensively reviewed and discussed between both authors in an effort to capture recurrent and prevalent themes across dyads and groups, while retaining focus on the individual and dyad voices. Interpretation and presentation of the findings was further reviewed and refined by all authors during the write-up stage.

## Findings

Five recurrent group themes were developed through cross-case analysis of interview data. Recurrent themes and subthemes are presented in Table 3 and illustrated below with quotes from participant interviews. Participants are referred to by pseudonym and family caregiver participants are marked with an asterisk.

**TABLE 3:** Recurrent group themes and subthemes from cross-case analysis of interview data.

Recurrent group themes	Group subthemes
Group TSW was an overwhelmingly positive shared experience, benefiting both members of the dyad and motivating further engagement with music	• Group TSW was a positive, fun and enjoyable experience • Dyads appreciated the opportunity to share the experience of TSW • Both members of the dyad experienced benefits, despite initial motivation to attend for the family member with dementia • Positive experience of group TSW highlighted its value, motivating dyads to “do more with music”
TSW was engaging and valuable as it stimulated mental processes and reignited participants’ interests, skills and memories	• Group TSW stimulated mental processes, including recollection of memories, creativity and communication • Value and enjoyment in the new, creative and stimulating aspects of TSW that sparked interest and motivated engagement • Group TSW reignited and connected participants with their skills and interests
TSW provided meaningful opportunities for reflection and connection with memories and life experiences	• Reminiscence “brought the past out of people” through storytelling and the creation of a song in the present • TSW gave space for participants to reflect on happy memories, life experiences and strengths • Experience of TSW positively impacted mood for some participants
TSW prompted interaction and collaboration, leading to social connections, empathic relationships and experiences of inclusion	• Positive social opportunity to build connections with others • Group TSW prompted interaction and collaboration as group members worked together toward the “one goal” • Group TSW fostered empathic relationships and experiences of inclusion
Group TSW included diverse challenges, however, the facilitated process supported participants to engage, highlighting abilities and challenging doubts	• Participants experienced diverse challenges but were able to “connect” and engage in different ways, which made it worthwhile • The approach and facilitation contributed to the achievability and success of the group TSW process • Participants contributed meaningfully, highlighting ability of self and others and challenging initial doubts, leading to feelings of pride and achievement

### Recurrent Theme 1. Group TSW Was an Overwhelmingly Positive Shared Experience, Benefiting Both Members of the Dyad and Motivating Further Engagement With Music

Group TSW was viewed as an overwhelmingly *positive, fun and enjoyable experience (subtheme 1)* that surpassed expectations. Numerous aspects of the experience were highlighted as enjoyable, including brainstorming, reminiscence, lyric creation, singing, and the song products or “what came out of it.” Participants used strong positive language to describe their experiences, with many stating that they “loved” the experience and found it very worthwhile, often despite initial apprehension or preconceptions.

“To be honest, I wasn’t that appreciative of it at first [laughing] […] It sounded really intimidating and challenging because I’ve never done anything like that. But once we got into it, I really enjoyed it […] I’m glad that I did it” (Carlo^∗^).

“I really loved it […] I thought it was lovely that you invited us to do this. It was something quite out of the blue. Yes, and it was so *good*” (Ann).

While some family caregivers were initially motivated to attend for the benefit of their family member with dementia, interview data indicated that dyads *appreciated the opportunity to share the experience (subtheme 2)* and *recognized benefits for both members of the dyad (subtheme 3)*. These included personal, social and relational benefits, as well as “flow on” benefits for family caregivers in seeing their family member and others in the group “happy.”

“I loved it […] as a thing that we could do together, that’s all I’m interested in […] I figured that it’s not for me, it’s for John, for John and the others for the music […] But of course, I’m getting benefit. Even if it’s just to know that John’s happy […] I mean, just doing things together is what it’s about. We’ve always done everything together and that’s the way I want to keep it” (Jan^∗^).

This appreciation of the opportunity to share the experience based on dyads’ existing relationship was echoed by other family caregivers, as well as participants with dementia who described how they “like being with” (Janet) their family member, are “happier” (Alan) when they are close to their family member, and that it was “more enjoyable” (Neil) attending together.

Others highlighted value in TSW as something they were able to “look forward to” and “go and do together” (Ali and Julia^∗^), which was described by some as a “unique experience” and “welcome change.”

“I haven’t really attended many groups with Isabella since she’s been ill, so it’s really been a unique experience, this one […] Especially now that I’m confined a lot, staying at home looking after Isabella, don’t get to get out as much anymore. Yeah, it was a welcome change” (Carlo^∗^).

Participants also differentiated group TSW from other types of engagement with music. Neil and Mei^∗^ recognized how the TSW process accentuated value “in the music,” where a focus on musical selves enabled them to learn more about each other and “interact more” (Neil). Carlo^∗^ further explained that the TSW group was different to recreational music experiences that had become inaccessible for the dyad.

“We’ve been to musicals and live shows and, at the start, Isabella was enjoying it. [But] as time went on you could see she was starting to disconnect and not relating to what’s going on. [TSW] is a different type of experience” (Carlo^∗^).

This *positive experience of group TSW highlighted its value, motivating dyads to* “*do more with music*” *(subtheme 4)*. Dyads stated that the group TSW program was something they were willing to commit time to, didn’t like stopping and that they would do again. Further, some described the experience as breaking a “barrier” to music engagement and giving them a new “appreciation” for music, as well as ideas for how to use music and songwriting beyond sessions.

“It certainly made me think about maybe looking at something else to do in relation with music […] I’ve never seen myself as any form of singer, but I think you break that barrier and you tend to not worry about that then […] It does open up that area for people that, it might be very good for them” (Dave^∗^), “Yes, we’ll do that again” (Lindy).

“It gave me an appreciation of music […] Last time I didn’t really appreciate music and now I do” (Neil), “And [songwriting] is a fun thing to do, which we can even try at home […] I didn’t know I could do, like, more with the music. I thought it was just a listening process. But now, after this experience, I find that we can do a lot more” (Mei^∗^).

### Recurrent Theme 2. TSW Was Engaging and Valuable as It Stimulated Mental Processes and Reignited Participants’ Interests, Skills and Memories

Participants described how group TSW *stimulated mental processes*, *including recollection of memories*, *creativity and communication (subtheme 1)*. One family caregiver described the effect of TSW on her husband as “making his brain sort of talk and express himself” (Mei^∗^), while for another participant with dementia, TSW felt like going “way back in your own brain” (Ann) where she was able to “link” thoughts, experiences and memories. Ann states:

“It did something to me, I can’t quite say […] It set me going […] It made my brain everything. Think about, all about what was and what might be and all this. It just made it move” (Ann).

Elizabeth^∗^ similarly described the creative method of song parody as “good to keep the brain moving,” while Jan^∗^ noticed how cognitive and creative aspects of TSW extended to her husband’s motivation to write poetry, and the comprehensibility of his writing, outside of sessions.

“After the first [session], I could see he had his thinking cap on, and he was writing […] After doing [TSW], he started, as you can see, he started to really come back to stuff that everybody could comprehend […] [And he was] *attempting* it more during that time” (Jan^∗^).

Importantly, dyads found *value and enjoyment in the new, creative and stimulating aspects of TSW that sparked interest and motivated engagement (subtheme 2)*. Dyads communicated an openness, curiosity and excitement about TSW as something they had “never done before” (Alan), and otherwise “never would have done” (Dave^∗^).

“For us, yes [it was worthwhile] because it was a new experience. Because we hadn’t done that, we wouldn’t have had the opportunity that we did have” (Tom), “I think it was a marvelous opportunity […] [speaking to Tom] I know what stood out for you – doing the recording. You were very intrigued with it all” (Ellen^∗^), “Yeah that’s right. That was something that was… I haven’t had that experience before. It was interesting” (Tom).

Value also came from the creative, cognitive and musical challenges within the TSW process.

“I was thrilled to bits with, with um, it was always coming and singing with these things […] I’d go out and get this and I’d start again […] [TSW was] creative plus difficult […] I *enjoyed* it, so it didn’t mean that I, I couldn’t do it […] I didn’t think I would [enjoy the challenge], but I did” (Ann).

For some, group TSW *reignited and connected them with their skills and interests (subtheme 3)*, including in language, poetry, music and singing. For example, John described how his longstanding enjoyment of writing poetry “flowed on” to TSW, while Ali was able to work with his pre-existing interest and ability with words through lyric writing and refinement. Ali further described a “positive spin-off” of the TSW program as it inspired poetic expression in his “choice of words” and “lyricism” when communicating with his wife outside of sessions.

For others, TSW reconnected them with their musical interests and backgrounds.

“[Speaking to Dawn] I remember toward the end of the last session, you were saying–It’s not often I get a chance to have, to sing…because in the past, you would get to sing every week, lots, lots of singing” (Ian^∗^). “Yeah, a couple of times a week, really, with groups” (Dawn). “Yeah, so you certainly said that last time, you know, you offered that up. So ah, yeah. You were enjoying it” (Ian^∗^).

### Recurrent Theme 3. TSW Provided Meaningful Opportunities for Reflection and Connection With Memories and Life Experiences

As well as being mentally and creatively stimulating, participants focused on how TSW connected them with memories and life experiences in a way that was personally meaningful and encouraged reflection.

Dyads noted that *reminiscence* “*brought the past out of people*” *through storytelling and the creation of a song in the present (subtheme 1)*. The TSW groups were an opportunity for “everyone” in the group to talk about “what they remembered, what was important to them” (Ian^∗^), with many describing the process as “taking you back,” “bringing back,” and “waking you up” to different memories and self-experiences. Participants with dementia expressed this as a “lovely” experience “because it all returns to you” (Ann) and reminds you that “there was a life back there” (Dawn), while for younger family caregivers it was an opportunity to learn “new information.”

Participants further explained the role of reminiscence in “bring[ing] the words *out* of their previous life” (George^∗^) and the here-and-now process of “building up something” (Ann) or making something new from these past experiences. One family caregiver described how this process led him to think of past experiences “in a different way.”

“With the picnic van one, back then that was […] a great day out and all the rest of it. But now when you look back on it, you go – wow! That was something special. In the context of *now*, you’re thinking – we don’t do those things anymore” (Ian^∗^).

Relatedly, dyads valued how *TSW gave space to reflect on happy memories, life experiences and personal strengths (subtheme 2)*. This was especially significant for participants who held “negative” perceptions of their present and future situations and environments or had limited existing meaningful opportunities for engagement and enjoyment. For example, one family caregiver from a residential aged care group described it being “better” that the group chose to write a song about their “previous life” as they would have had “difficulty writing a song about this, living in this [environment]” (George^∗^). A participant with dementia living in residential care described a similar experience:

“Being in a place like this, [TSW] helps me to sort of forget about all this, you know. Because this is not where I want to be, of course. And I’m going to be in here for the rest of my life […] But that’s why [TSW] helps me as much as going back […] you go back and you think, oh, yeah, when we did this or whatever [Michelle^∗^: Of happy times]. Yeah, it’s lovely. Yeah, absolutely. And especially for us now, our times. You know, we don’t know how long we’re going to be around and so, you know, to go back to all these things […] It’s very special” (Ann).

This *experience of TSW positively impacted mood for some participants (subtheme 3)*. Ann described *feeling* like her younger, happier and more confident self during TSW, explaining: “When I was doing that, I was being that person, that girl […] I’m sort of two people. Like now, I think I’m very ordinary, down there, and before, I thought I was really good, happy.” Ann’s daughter similarly highlighted the significance of TSW because of “how happy it made Mum” (Michelle^∗^).

“[Speaking to Ann] You don’t always have a lot of things that make you happy. So, it was nice to see you enthusiastic about doing something and coming back feeling good in yourself [Ann: Yeah, well I agree with that] rather than coming back and, oh yeah, that was nice and forget about it. You know, if I said that we were going […] we were almost running down there!” (Michelle^∗^).

Carlo^∗^, who was caring for his wife at home, also identified TSW as having a positive impact on his mood. He described how TSW prompted reflection on his “whole” life’ and the dyad’s “journey together,” which helped him find positives, rather than getting caught in a “downward spiral.”

“[TSW] forced me to think about things and reflect on stuff. Just reflect on what’s happening with us now, with Isabella and I […] Sometimes you can get caught in the moment when things aren’t going well and it’s a downward spiral from there. Yeah, but this helped me sort of reflect and look at some positives […] We’ve had an amazing life, I have to say. We’ve done lots of amazing things together […] and a lot of the stuff that we did, we wouldn’t have been able to do if it wasn’t for Isabella […] [It was meaningful to reflect on] our journey together. Just what our visions were when we were young, what we wanted to do, and the fact that we worked toward them and achieved them” (Carlo^∗^).

### Recurrent Theme 4. TSW Prompted Interaction and Collaboration, Leading to Social Connections, Empathic Relationships and Experiences of Inclusion

Dyads highlighted clear benefits related to the group aspect of the program. Firstly, participants described group TSW as a *positive social opportunity to build connections with others (subtheme 1)*, which sometimes continued outside of sessions or even following the program. This opportunity to get to know others in a similar situation was relevant in both community and residential care groups. In fact, many residents and family members living in and visiting the same care home had either not met before or only knew each other as acquaintances.

“It was enjoyable working with the others. You know, sort of got to know, well I had met Ellen and George before, but I sort of got to know them a little bit better” (Elizabeth^∗^).

Participants described TSW as *prompting interaction and collaboration as group members worked together toward the* “*one goal*” *(subtheme 2)*. Dyads highlighted this as an advantage of group TSW, differentiating it from other group experiences. Participants attributed this to the music, and the shared goal and collective effort of writing a song, which meant that song creations felt like they were “all of everyone” (Dave^∗^).

“[What I found meaningful was] just the way everyone has to put in to make the song. Yeah, so, it’s like interactive […] it’s got a sort of, a flavor to it. So it’s got your experiences, Carlo’s experiences, and everyone’s” (Mei^∗^).

Of working as a group, Neil stated that he “enjoyed the experience” as the other dyad “had different ideas from the ones we had.” Others further explained the collaborative nature of TSW:

“I’d say something and Ian would say something and Mum would say something or Dawn would say something and it just sort of jelled. I think we were a good mix […] we cooperated really well […] everyone allowed everyone a chance to speak” (Michelle^∗^). “One of us would come up with something and then that would set off the rest of us” (Ann).

Participants also described the TSW group process as involving sharing something of yourself and accepting others as they were, which *fostered empathic relationships and experiences of inclusion (subtheme 3)*.

“You have to really make room for the others to get their ideas and all that […] but it’s nice! It’s nice to express a group, to express other people’s ideas or, yeah, to listen, to be listened to, to share. It’s nice, it’s a different experience”.

This group process encouraged participants to think about others in an empathic way. Carlo^∗^ described listening to and taking in other people’s experiences during TSW and how this encouraged him to “think about others and what they may go through.” Other participants, in both community and residential care contexts, described experiences of connection and inclusion resulting from the “underlying understanding” and respect between group members who were all experiencing their own challenges.

“It was good having the others there, too. Because you could see they’re all…they’ve got their challenges too, didn’t they? […] So you’re not, sort of like, you’re the odd one out type thing. And everybody just accepted everybody as they were, which was lovely” (Elizabeth^∗^).

“[We’re] people with similar problems. So there’s an underlying understanding between everybody. There’s an openness. It’s a safe space where you can, you know, share, talk, do things and not worry about what would people think, how they would judge or whatever if we do something silly” (Julia^∗^).

### Recurrent Theme 5. Group TSW Included Diverse Challenges, However, the Facilitated Process Supported Participants to Engage, Highlighting Abilities and Challenging Doubts

Participants described the TSW groups as including people with different personalities, abilities and needs and noted challenges related to adjusting to the group context and new environment, maintaining focus, communicating ideas, and self-consciousness or lack of confidence in ability to contribute. However, interview data indicated that, *while participants did experience diverse challenges in participating in group TSW, they were also able to* “*connect*” *and engage in different ways, which made it worthwhile (subtheme 1)*.

“It varies in terms of the ability, you know […] It’s hard to maintain their, no not maintain their interest. I think their interest is there, but the mind…focus, switch off and on […] It’s not an easy thing to get [the group] all participating at the same time. But I thought that was exceptional, what was got out of the sessions” (George^∗^).

A number of participants communicated that there was “space” for group members to communicate and engage in various ways. Ali described that, although communicating his ideas was challenging due to speech difficulties, “space was available all the time” and he felt his ideas and contributions were included in the group’s song creations. Carlo^∗^ also explained how his wife, who did not communicate using verbal language and was unable to contribute directly to lyric creation, was “very intrigued” and “very connected” during some sessions, particularly during the use of live music and visual resources. This contrasted to Carlo’s description of Isabella “disconnecting” and “not relating” during past music experiences (such as attending concerts and musicals) and was supported by the observations of other group members.

“Although Isabella doesn’t talk a lot, I find she’s getting more interested in it. Toward the end she was reaching out, even to Neil […] So she’s more, like expressing herself more. Cause the first time I noticed when she came, she was very quiet […] But toward the end, she was sort of getting more outgoing” (Mei^∗^).

Participants also identified that *the approach and facilitation contributed to the achievability and success of the group TSW process (subtheme 2)*, supporting participants to engage and making songwriting surprisingly accomplishable for groups who had never written a song before. Participants described how the music therapist’s musical skills supported the group’s songwriting endeavors, and how the TSW process itself “flowed” much better than expected. Dyads also valued the facilitators’ “gentle” and “relaxed” approach, leadership and encouragement, and patience in “managing” group dynamics and the different needs of group members.

“I thought it was gonna be really tough. I thought – I can’t write songs, […] how is this going to work? But [the music therapist] led the group well and encouraged people to have a go. And, you know, you think about what um, the topic was, and what they remembered from those days and…I just thought that everybody seemed happy and relaxed and enjoyed themselves” (Michelle^∗^).

Further, participants recognized that *group members contributed meaningfully, highlighting ability of self and others and challenging initial doubts, leading to feelings of pride and achievement (subtheme 3)*. As most participants held beliefs about the difficulty of songwriting and their capacity to contribute, many were “surprised” by their own abilities when pushed outside their “comfort zones.”

“I don’t think I believed that we could write any words […] And it was a surprise to me that I actually came up with a few ideas. I like to – as a rule – I like to sit in the background and just listen […] so yeah, it was a big surprise to me. A big surprise” (Jan^∗^).

Neil also described a personally meaningful part of the program as “learning to express myself.” His wife, Mei^∗^ directly attributed this to the facilitated TSW experience, which encouraged participants to “voice” their ideas and opinions.

“He’s quite quiet normally in the group. So, with this music therapy thing, he’s talking a bit more. Maybe because [the music therapist is] asking his opinion […] Cause in the walking group you just sit there and you won’t talk. Because no one asks you to talk, you won’t talk. Whereas here, you’re asked to voice your opinion. You have to give something” (Mei^∗^).

Participants also recognized the contributions of others and the collective achievement of the whole group. Ellen^∗^ described there being “more input” from group members than she thought there might be, while Ali felt “proud of the effort of the group.” Other family caregivers specifically highlighted the integral roles and contributions of participants with dementia, explaining that “without their ideas, we wouldn’t have been able to do it” (Michelle^∗^).

Finally, participants articulated meaning in being able to see the results of the groups effort and contributions, which led to a “sense of achievement’ (Mei^∗^) and ‘satisfaction [in] knowing it had come together” (Ellen^∗^).

“I think maybe how productive we were once we got rolling […] that’s the thing that stuck out for me the most […] You’re engaged in this and, and it’s meaningful because you’re contributing to it [Dawn: You’re doing it!] and seeing the results of it later […] I seem to remember in the last session we were all pretty chuffed with what we had done’ (Ian^∗^), ‘Oh yeah. We’d all, we’d all put in” (Dawn).

## Discussion

This study aimed to explore how people with dementia and their family caregivers experienced a 6-week group TSW program attended together as dyads. Dyads shared spousal or parent-adult child relationships, lived together or separately, and had diverse abilities, needs, interests, musical backgrounds and ways of communicating. Five recurrent group themes were developed through interpretative phenomenological analysis of participant interviews, highlighting collaborative, relational, dyadic, and personal benefits of group TSW with dyads.

The collaborative group aspect of TSW fostered social connections, empathic relationships and experiences of inclusion (theme 4). These experiences of empathy, belonging and affinity with others who have similar lived experience are reflected in community-based music therapy research on therapeutic group singing with dyads (Clark et al., 2018), and group TSW with family caregivers (Baker and Yeates, 2018; Baker et al., 2018) and people with dementia (Baker and Stretton-Smith, 2017). In residential aged care contexts, non-cohabiting family caregivers of people with dementia have also highlighted value in the mutual support and empathy found within carer support groups (Hennings and Froggatt, 2019). This emphasis on mutual, empathic support and understanding, explained in the current study as inclusive and non-judgmental groups where everyone “accepted” each other as they were, suggests that similar experiences may not be readily available in other social contexts. These findings are consistent with research on public-stigma in the context of dementia, reporting that lack of knowledge, stereotypes, negative beliefs and discrimination are common among the general public toward people with dementia and their family caregivers (Nguyen and Li, 2020). It is possible that empathic group connections, as experienced by participants in the current study, may help to mitigate experiences of public or social stigma (Gorska et al., 2018).

Participants in our study recognized the role of music in promoting connection and interaction, differentiating group TSW from other group experiences. This finding is supported by research informing the psychosocial model of music in dementia, indicating that meaningful musical experiences foster emotional and interpersonal connectedness, and that the ability to develop and maintain musical relationships is an ongoing resource for people with dementia despite physical or cognitive decline (McDermott et al., 2014). Our findings, and those of Clark et al. (2018), further recognize how a common musical goal or purpose (singing or creating a song) prompted collaboration and brought dyads together. However, consistent with theory on group TSW (Baker, 2015), participants in the current study further differentiated group TSW as an opportunity “to listen, to be listened to, to share” and “express a group” through writing a song. Group process and community music therapy models, drawn on in an effort to invite participation and empower the voices of individuals and dyads toward a collective effort (Yalom and Leszcz, 2005; Stige and Aarø, 2012), were also reflected in participants’ described experiences. Our findings recognized that participants were able to contribute in diverse ways, received space and encouragement to voice their opinion, and worked collaboratively toward song products that were “all of everyone.”

Dyads’ descriptions of group TSW as a positive *shared* experience (theme 1) is reflected in group therapeutic singing research with community-dwelling dyads (Clark et al., 2018). Participants in the current study explained how attending TSW sessions as a dyad was a “unique opportunity” to meaningfully engage with music together. This was especially important for dyads for whom other group or music experiences had become inaccessible. Further, TSW helped dyads appreciate how they could “do more with music,” from engaging in songwriting together at home to seeking similar opportunities for group music participation. Community-dwelling family and spousal dyads in the Clark et al. (2018) study similarly described how therapeutic singing groups enhanced accessibility to music, leading to positive personal and relational experiences. The current study extends these findings to residential care contexts, where family and spousal dyads described group TSW as a “bonding” experience, that was simultaneously new and different, and drew on their existing relationship with each other and with music. In residential aged care, activities and interventions that support person and relationship-centered care can encourage involvement of non-cohabiting family caregivers (Hennings and Froggatt, 2019), which in turn increases the engagement levels of family members with dementia (Milne, 2011). In the current study, benefits were described for both members of the dyad, including positive impacts for family caregivers from observing their family member engaged, happy and motivated to participate in group TSW. Regardless of context, interventions that offer meaningful opportunities for togetherness, as described by participants in the current study, are recommended to support connection and relationship quality (Wadham et al., 2016).

Participants particularly valued opportunities to reflect and connect with the past during TSW, involving recognition of personal and shared memories, strengths and life experiences (theme 3). These findings resonate with notions of couplehood and family centeredness where partnerships built over a lifetime are acknowledged and appreciated within the current relationship story (Wadham et al., 2016; Hao and Ruggiano, 2020). Interventions that support dyads to explore their shared life together through storytelling may promote long standing feelings of reciprocity, connection, and resilience, which families and couples can draw on to support emotional and psychological wellbeing as they navigate challenging experiences (Conway et al., 2018). For example, in the current study, one family caregiver described TSW sessions as making him reflect on his “whole life” and “journey together” with his wife in a way that supported him to “focus on the positives” rather than getting caught in a “downward spiral.” These findings also resonate with previous research on group TSW with family caregivers who differentiated TSW from traditional carer support groups in that they were able to reflect on, and gain clarity and insight into the “whole carer journey,” and look for positives in their experiences rather than only focusing on everyday stressors and challenges (Baker et al., 2018). This indicates that, for some, TSW may stimulate this kind of reflection and exploration within and beyond sessions, regardless of whether groups include dyads or family caregivers only. Further, this experience had particular meaning for some participants in residential care contexts, who valued being able to connect with and tell stories about their past, rather than the current situation. One resident described how this enabled her to remember and feel like her younger, happier and more confident self, resonating with previous research suggesting residents with dementia actively seek to cope with their current situations by affirming their past sense of self and identity (Milne, 2011).

As well as the emotional experience of connecting to and creating something new from past memories and experiences, participants described group TSW as a creative and cognitively stimulating process (theme 2). In our study, and previous TSW research with people with dementia (Baker and Stretton-Smith, 2017), participants explained how the mental stimulation of creating a song was particularly valuable because they learnt new skills or were able to build on existing interests and abilities. Both participants with dementia and family caregivers in our study further described positive impacts of TSW on verbal and written communication and expression and ability to “link” and recall “forgotten” memories. These findings are further supported by TSW research with people with advanced dementia living in aged care, which demonstrated improved cognition, language, orientation and memory following a 16-week TSW program (Hong and Choi, 2011). While the use of music is known to support people with dementia to access autobiographical memories (Cuddy et al., 2015), musical skills and associated knowledge (Spiro, 2010; Jacobsen et al., 2015), group TSW further extends the cognitive and creative benefits of music-making and listening through brainstorming with others to create lyrics and musical ideas (Baker, 2015). These benefits from group TSW align with recommendations from systematic reviews, recognizing how people with dementia seek opportunities for continued growth, skill development and engagement in new and meaningful activities (Wolverson et al., 2016; Low et al., 2017), and family caregivers are looking for ways to support both their own and family member’s psychological health and wellbeing (McCabe et al., 2016; Egilstrod et al., 2019).

Participants explained individual challenges associated with group TSW, while also recognizing that the “way it’s done” supported contributions from all group members, allowing abilities to shine and overcoming initial doubts (theme 5). Some of the challenges experienced are reflected in previous group TSW research with people with dementia, including participant feelings of self-consciousness and perceived lack of ability to contribute (Baker and Stretton-Smith, 2017). This may be understood in relation to “self-stigma” where people with dementia internalize stigma and negative beliefs about the self, which may manifest in feelings of grief, loss of confidence and depression (Nguyen and Li, 2020). For example, one participant with dementia described feeling like “two people,” comparing her perception of her past self (“really good, happy”) with her present self (“very ordinary, down there”). However, in the current and previous research, participants with dementia also described how group TSW facilitated by a music therapist promoted meaningful contributions from participants and offered opportunities for success, resulting in feelings of pride and achievement. These experiences of accomplishment and ability may help to shift attitudes and promote positive and constructive self-beliefs of people with dementia, contributing to wellbeing and quality of life (Nguyen and Li, 2020). Further, in the current study, the perceived difficulty of songwriting and surprise at ability to contribute was shared by family caregivers, suggesting these responses were also related to preconceptions about musicality and creativity. Our findings are similarly represented in previous research on group TSW with family caregivers, who described their song creations as exceeding expectations after initially doubting they would have something to contribute or feeling insecure about their perceived lack of musical ability (Baker and Yeates, 2018). Participants in the current study also recognized how the music therapists encouraged and supported individuals to contribute meaningfully to the group product. These findings help to explain how concepts of personhood, which have previously been applied to therapeutic group singing (Clark et al., 2018), also promote individual engagement and collaborative achievement among group TSW participants (Baker and Stretton-Smith, 2017).

### Limitations

As an interpretative phenomenological analysis study, the analysis of ten interviews involving nineteen participants could be considered large. Although there is no rule regarding participant numbers, larger sample sizes are less common due to the idiographic focus and purpose of interpretative phenomenological analysis, which aims to explore participants’ lived experiences and meaning making in-depth and create rich descriptions that “give full appreciation to each participant’s account” (Pietkiewicz and Smith, 2014, 9). In the current study, while the analysis was time consuming, authors engaged in an iterative and reflexive interpretative phenomenological analysis process, and we believe findings authentically represent and value participants’ descriptions of their experiences.

There were some further limitations with using interviews to collect qualitative data. While most participants were able to contribute through interviews, one participant with dementia from the primary study was unable to take part as she did not communicate using verbal language and others found it challenging. However, consistent with previous music therapy research, the authors did consider how interviews could be conducted to reduce challenges and support participants to be involved in the research process, including the use of dyadic interviews and dual role of the music therapists/interviewers (McDermott et al., 2014; Clark et al., 2018; Thompson et al., 2020). Considerations around trust and rapport, using prompts, and sensitivity and awareness to all verbal, non-verbal and non-behavioral communication are important factors in facilitating semi-structured interviews (Pietkiewicz and Smith, 2014) and need to be carefully considered and extended to support the participation of people with dementia in qualitative research.

Finally, the broad inclusion criteria may be considered a limitation. The current study included dyads living together at home or separately because the person with dementia lived in a residential aged care facility. We also recruited dyads who shared either spousal or parent-adult child relationships. It is likely that these differing contextual and relationship factors would have impacted how participants experienced the group TSW process. However, as there is no previous research on group TSW with dyads, we sought to gain an understanding from diverse perspectives with the intention of drawing on findings to inform more focused research in the future. We also took care to attribute quotations and highlight the perspectives of dyads across groups.

### Recommendations

Future research may wish to further explore the diverse and unique experiences of cohabiting and non-cohabiting spousal and family dyads in community and residential care contexts. Previous research on dyad-based therapeutic singing groups has taken place in community settings (Clark et al., 2018) and, as indicated by existing literature, there are few psychosocial or therapeutic group interventions designed for both people with dementia and their families in residential care settings (Rausch et al., 2017). Further, the first-person experiences of people in later stages of dementia (Low et al., 2017), and family caregivers of people with dementia living in residential care, are often not included in the research (Hennings and Froggatt, 2019). Future research might examine how dyads across these different contexts and relationships experience group TSW.

We sought to clearly describe a theoretical approach to group TSW that was designed to meet the unique needs and promote the diverse strengths, resources, interests and contributions of group members, while taking into account the voice of the individual, shared voice of the dyad and collective voice of the songwriting group. McDermott et al. (2014) argued the importance of identifying relevant theoretical frameworks to inform music therapy research with people who have dementia in order to contextualize findings and understand the mechanisms behind how and why music interventions may benefit psychosocial wellbeing. A previous music therapy study that utilized a dyadic approach (Clark et al., 2018) has been acknowledged in recent research promoting the development of family centeredness in dementia care (Hao and Ruggiano, 2020). As such, future music therapy research may wish to more overtly draw on family centered care models specific to dementia care.

Finally, the capacity of people with dementia to contribute their perspectives through interviews, and the importance of including their voices in the research concerning them, has been highlighted in the literature (Low et al., 2017; Thompson et al., 2020). At the same time, it is important to consider the limitations of interviews in research with people whose abilities and preferences for ways of interacting and communicating may not be through verbal language. As our approach to group TSW offered a range of ways of contributing to and engaging in the intervention, other artistic or creative mediums for expression, such as contributions through songwriting or music-making, may promote personhood and better capture the voices and lived experience of people with dementia in research.

## Conclusion

There is limited research on group music therapy interventions, and to our knowledge no existing literature on group TSW, with people living with dementia and family caregiver dyads. The current study draws together recent research indicating value in TSW groups with people with dementia and family caregivers participating separately, and therapeutic singing groups with people with dementia and family caregiver dyads, therefore contributing significant and novel findings regarding the experience and meaning of group TSW with dyads. Our study also works to further acknowledge and prioritize the voices of people with dementia and their families in the research concerning them. While aspects of the group TSW process presented challenges for group members, participants described it as an overwhelmingly positive and worthwhile experience offering diverse opportunities for engagement based on participants’ abilities, needs and interests. Dyads emphasized group TSW as a “unique” and “meaningful” group experience that held value because they could attend together. We also found that the considered approach was reflected in participants’ described experiences, emphasizing that people with diverse abilities, skills, and styles of communication were able to engage in group TSW together, whether through lyric creation and storytelling, musical contributions, or enjoyment and connection in the moment, leading to positive personal, dyadic and empathic group experiences. These findings highlight the value of creative dyad-based therapeutic group interventions, such as TSW, that are informed by family centered or relational perspectives and emphasize personhood and couplehood of individuals and dyads within a group. In addition, our inclusion of spousal dyads living together at home and spousal and family dyads living separately because the person with dementia was living in residential care, contributes to an understanding of how dyad connectivity is experienced and can be supported within different relationships and contexts and through different phases of the disease trajectory.

## Data Availability Statement

The original contributions presented in the study are included in the article/supplementary material, further inquiries can be directed to the corresponding author/s.

## Ethics Statement

The studies involving human participants were reviewed and approved by the Education Fine Arts Music & Business Human Ethics Sub-Committee, University of Melbourne. The patients/participants provided their written informed consent to participate in this study.

## Author Contributions

IC was responsible for the study design and project management. PS-S facilitated the intervention and conducted qualitative interviews. IC and PS-S jointly analyzed the interview data and drafted the manuscript. JT assisted with conceptualization of the project design, acted as an advisor throughout the project, and reviewed the manuscript. AC facilitated some intervention sessions and interviews and reviewed the manuscript. FB and Y-EL provided general support during the project and contributed to the final manuscript. All authors contributed to the article and approved the submitted version.

## Conflict of Interest

The authors declare that the research was conducted in the absence of any commercial or financial relationships that could be construed as a potential conflict of interest.

## References

[B1] Ablitt A. Jones G. V. Muers J. (2009). Living with dementia: a systematic review of the influence of relationship factors. *Aging Ment. Health* 13 497–511. 10.1080/13607860902774436 19629774

[B2] Baker F. A. (2015). *Therapeutic Songwriting: Developments in Theory, Methods, and Practice.* New York: Palgrave Macmillan.

[B3] Baker F. A. (2017). A theoretical framework and group therapeutic songwriting protocol designed to address burden of care, coping, identity, and wellbeing in caregivers of people living with dementia. *Aust. J. Music Ther.* 28 16–33.

[B4] Baker F. A. Stretton-Smith P. A. (2017). Group therapeutic songwriting and dementia: exploring the perspectives of participants through interpretative phenomenological analysis. *Music Ther. Perspect.* 36 50–66. 10.1093/mtp/mix016

[B5] Baker F. A. Yeates S. (2018). Carers’ experiences of group therapeutic songwriting: an interpretive phenomenological analysis. *British Journal of Music Therapy* 32, 8–17. 10.1177/1359457517728914

[B6] Baker F. A. Grocke D. Pachana N. A. (2012). Connecting through music: a study of a spousal caregiver- directed music intervention designed to prolong fulfilling relationships in couples where one person has dementia. *Aust. J. Music Ther.* 23 4–21.

[B7] Baker F. A. Stretton-Smith P. A. Clark I. N. Tamplin J. Lee Y.-E. (2018). A group therapeutic songwriting intervention for family caregivers of people living with dementia: a feasibility study with thematic analysis. *Front. Med.* 5:151. 10.3389/fmed.2018.00151 PMC5972290 29872659

[B8] Brotons M. Marti P. (2003). Music therapy with Alzheimer’s patients and their family caregivers: a pilot project. *J. Music Ther.* 40 138–150. 14505442 10.1093/jmt/40.2.138

[B9] Clair A. A. (2002). The effects of music therapy on engagement in fmaily caregiver and care receiver couples with dementia. *Am. J. Alzheimer’s Dis. Dement.* 17 286–290. 10.1177/153331750201700505 12392265 PMC10833679

[B10] Clair A. A. Ebberts A. G. (1997). The effects of music therapy on interactions between family caregivers and their care receivers with late stage dementia. *J. Music Ther.* 34 148–164. 10.1093/jmt/34.3.148

[B11] Clark I. N. Stretton-Smith P. A. Baker F. A. Lee Y. E. C. Tamplin J. (2020). “It’s feasible to write a song”: a feasiblity study examining group therapeutic songwriting for people living with dementia and their family caregivers. *Front. Psychol.* 11:1951. 10.3389/fpsyg.2020.01951 PMC7426520 32849143

[B12] Clark I. N. Tamplin J. Baker F. A. (2018). Community-dwelling people living with dementia and their family caregivers’ experiences of therapeutic group singing: a qualitative thematic analysis. *Front. Psychol.* 9:1332. 10.3389/fpsyg.2018.01332 PMC6077620 30104994

[B13] Conway E. R. Watson B. Tatangelo G. McCabe M. (2018). Is it all bleak? a systematic review of factors contributing to relationship change in dementia. *Int. Psychogeriatr.* 30 1619–1637. 10.1017/s1041610218000303 29667571

[B14] Cuddy L. L. Sikka R. Vanstone A. (2015). Preservation of musical memory and engagement in healthy aging and Alzheimer’s disease. *Ann N Y Acad Sci* 1337 223–231. 10.1111/nyas.12617 25773638

[B15] Egilstrod B. Ravn M. B. Petersen K. S. (2019). Living with a partner with dementia: a systematic review and thematic synthesis of spouses’ lived experiences of changes in their everyday lives. *Aging Ment Health* 23 541–550. 10.1080/13607863.2018.1433634 29405735

[B16] Fusar-Poli L. Bieleninik L. Brondino N. Chen X. J. Gold C. (2018). The effect of music therapy on cognitive functions in patients with dementia: a systematic review and meta-analysis. *Aging Ment Health* 22 1097–1106. 28691506 10.1080/13607863.2017.1348474

[B17] García-Valverde E. Badia M. Begoña Orgaz M. Gónzalez-Ingelmo E. (2019). The influence of songwriting on quality of life of family caregivers of people with dementia: an exploratory study. *Nord. J. Music Ther.* 29 4–19. 10.1080/08098131.2019.1630666

[B18] Gorska S. Forsyth K. Maciver D. (2018). Living with dementia: a meta-synthesis of qualitative research on the lived experience. *Gerontologist* 58 e180–e196. 28069886 10.1093/geront/gnw195 PMC5946830

[B19] Hanser S. B. Butterfield-Whitcomb J. Kawata M. Collins B. E. (2011). Home-based music strategies with individuals who have dementia and their family caregivers. *J. Music Ther.* 48 2–27. 10.1093/jmt/48.1.2 21866711

[B20] Hao Z. Ruggiano N. (2020). Family-centeredness in dementia care: what is the evidence? *Soc. Work Health Care* 59 1–19. 10.1080/00981389.2019.1690089 31900066

[B21] Hellström I. Nolan M. Lundh U. (2007). Sustaining ‘couplehood’: spouses’strategies for living positively with dementia. *Dementia* 6 383–409. 10.1177/1471301207081571

[B22] Hennings J. Froggatt K. (2019). The experiences of family caregivers of people with advanced dementia living in nursing homes, with a specific focus on spouses: a narrative literature review. *Dementia* 18 303–322. 10.1177/1471301216671418 27856694

[B23] Hiller J. (2016). “Epistemological foundations of objectivist and interpretivist research,” in *Music Therapy Research*, eds Wheeler B. Murphy K. (Dallas, TX: 236-268).

[B24] Hong I. S. Choi M. J. (2011). Songwriting orientated activities improve the cognitive functions of the aged with dementia. *Arts Psychother.* 38 221–228. 10.1016/j.aip.2011.07.002

[B25] Jacobsen J. H. Stelzer J. Fritz T. H. Chetelat G. La Joie R. Turner R. (2015). Why musical memory can be preserved in advanced Alzheimer’s disease. *Brain* 138(Pt 8), 2438–2450. 10.1093/brain/awv135 26041611

[B26] Keady J. D. (1999). *The Dynamics of Dementia: a Modified Grounded Theort Study. Doctor of Philosophy, School of Nursing, Midwifery and Health Studies, University of Wales.*

[B27] Kitwood T. (1997). *Dementia Reconsidered: The Person Comes First.* Milton Keynes: Open University Press.

[B28] Klein C. M. Silverman M. J. (2012). With love from me to me: using songwriting to teach coping skills to caregivers of those With Alzheimer’s and other dementias. *J. Creat. Ment. Health* 7, 153–164. 10.1080/15401383.2012.685010

[B29] Li H. C. Wang H. H. Lu C. Y. Chen T. B. Lin Y. H. Lee I. (2019). The effect of music therapy on reducing depression in people with dementia: a systematic review and meta-analysis. *Geriatr. Nurs.* 40 510–516. 10.1016/j.gerinurse.2019.03.017 31056209

[B30] Low L.-F. Swaffer K. McGrath M. Brodaty H. (2017). Do people with early stage dementia experience prescribed disengagement? a systematic review of qualitative studies. *Int. Psychogeriatr.* 30 807–831. 10.1017/s1041610217001545 28828999

[B31] McCabe M. You E. Tatangelo G. (2016). Hearing their voice: a systematic review of dementia family caregivers’ needs. *Gerontologist* 56 e70–e88. 27102056 10.1093/geront/gnw078

[B32] McDermott O. Crellin N. Ridder H. M. Orrell M. (2013). Music therapy in dementia: a narrative synthesis systematic review. *Int. J. Geriatr. Psychiatry* 28 781–794. 10.1002/gps.3895 23080214

[B33] McDermott O. Orrell M. Ridder H. M. (2014). The importance of music for people with dementia: the perspectives of people with dementia, family carers, staff and music therapists. *Aging Mental Health* 18 706–716. 10.1080/13607863.2013.875124 24410398 PMC4066923

[B34] McGillick J. Murphy-White M. (2016). “Experiences and perspectives of family caregivers of the person with dementia,” in *Dementia Care: an Evidence-Based Approach*, eds Boltz M. Galvin J. E. (Cham: Springer), 198–214.

[B35] Milne A. (2011). Living with dementia in a care home: capturing the experiences of residents. *Qual. Ageing Older Adults* 12 76–85. 10.1108/14717791111144687

[B36] Nguyen T. Li X. (2020). Understanding public-stigma and self-stigma in the context of dementia: a systematic review of the global literature. *Dementia* 19 148–181. 10.1177/1471301218800122 31920117

[B37] Pietkiewicz I. Smith J. A. (2014). A practical guide to using interpretative phenomenological analysis in qualitative research psuchology. *Psychol. J.* 20 7–14.

[B38] Rausch A. Caljouw M. A. van der Ploeg E. S. (2017). Keeping the person with dementia and the informal caregiver together: a systematic review of psychosocial interventions. *Int. Psychogeriatr.* 29 583–593. 10.1017/s1041610216002106 27890029

[B39] Silber F. Hes J. (1995). The use of songwriting with patients diagnosed with Alzheimer’s disease. *Music Ther. Perspect.* 13 31–34. 10.1093/mtp/13.1.31

[B40] Smith J. A. Flowers P. Larkin M. (2009). *Interpretative Phenomenological Analysis: Theory, Method and Research.* London: Sage.

[B41] Spiro N. (2010). Music and dementia: observing effects and searching for underlying theories. *Aging Ment. Health* 14 891–899. 10.1080/13607863.2010.519328 21069595

[B42] Stige B. Aarø L. E. (2012). *Invitation to Community Music Therapy.* New York: Routledge.

[B43] Tamplin J. Clark I. N. (2019). “Therapeutic music interventions to support people with dementia living at home with their family caregivers,” in *Music and Dementia: From Cognition to Therapy*, eds Baird A. Garrido S. Tamplin J. (New York: Oxford University Press), 269–287. 10.1093/oso/9780190075934.003.0013

[B44] Tamplin J. Clark I. N. Lee Y. C. Baker F. A. (2018). Remini-sing: a feasibility study of therapeutic group singing to support relationship quality and wellbeing for community-dwelling people living with dementia and their family caregivers. *Front. Med.* 5:245. 10.3389/fmed.2018.00245 PMC6127293 30234118

[B45] Thompson Z. Baker F. Clark I. N. Tamplin J. (2020). “Inclusive music therapy research: an exploration into inclusive qualitative data collection practices to support participants living with dementia,” in *Proceedings of the 16th World Congress of Music Therapy. Special Issue of Music Therapy Today*, Vol. 16, eds Mercadal-Brotons M. Heiderscheit A. 56–57. Available online at: http://musictherapytoday.wfmt.info

[B46] Unadkat S. Camic P. M. Vella-Burrows T. (2017). Understanding the experience of group singing for couples where one partner has a diagnosis of dementia. *Gerontologist* 57 469–478. 26783138 10.1093/geront/gnv698

[B47] van der Steen J. T. Smaling H. J. A. van der Wouden J. C. Bruinsma M. S. Scholten R. J. P. M. Vink A. C. (2018). Music-based therapeutic interventions for people with dementia. *Cochrane Database Syst. Rev.* 7:CD003477. 10.1002/14651858.CD003477.pub4 PMC6513122 30033623

[B48] Wadham O. Simpson J. Rust J. Murray C. (2016). Couples’ shared experiences of dementia: a meta-synthesis of the impact upon relationships and couplehood. *Aging Ment. Health* 20 463–473. 10.1080/13607863.2015.1023769 25811103

[B49] Wolverson E. L. Clarke C. Moniz-Cook E. D. (2016). Living positively with dementia: a systematic review and synthesis of the qualitative literature. *Aging Ment. Health* 20 676–699. 10.1080/13607863.2015.1052777 26078084

[B50] Yalom I. Leszcz M. (2005). *Theory and Practice of Groyp Psychotherapy*, 5th Edn. New York: Basic Books.

